# Supplementary material for: The interactome of intact mitochondria by cross-linking mass spectrometry provides evidence for coexisting respiratory supercomplexes
Source: Mol Cell Proteomics. 2017 Dec 8;17(2):216–32. doi: 10.1074/mcp.RA117.000470 (PMC5795388; doi:10.1074/mcp.RA117.000470)
Supplement: Supplemental Data [file supp_RA117.000470_133922_0_supp_23978_fzffwf.zip › spectra_annotation/mito_spectra_annotation/53-1-3-1-12-1.pdf]

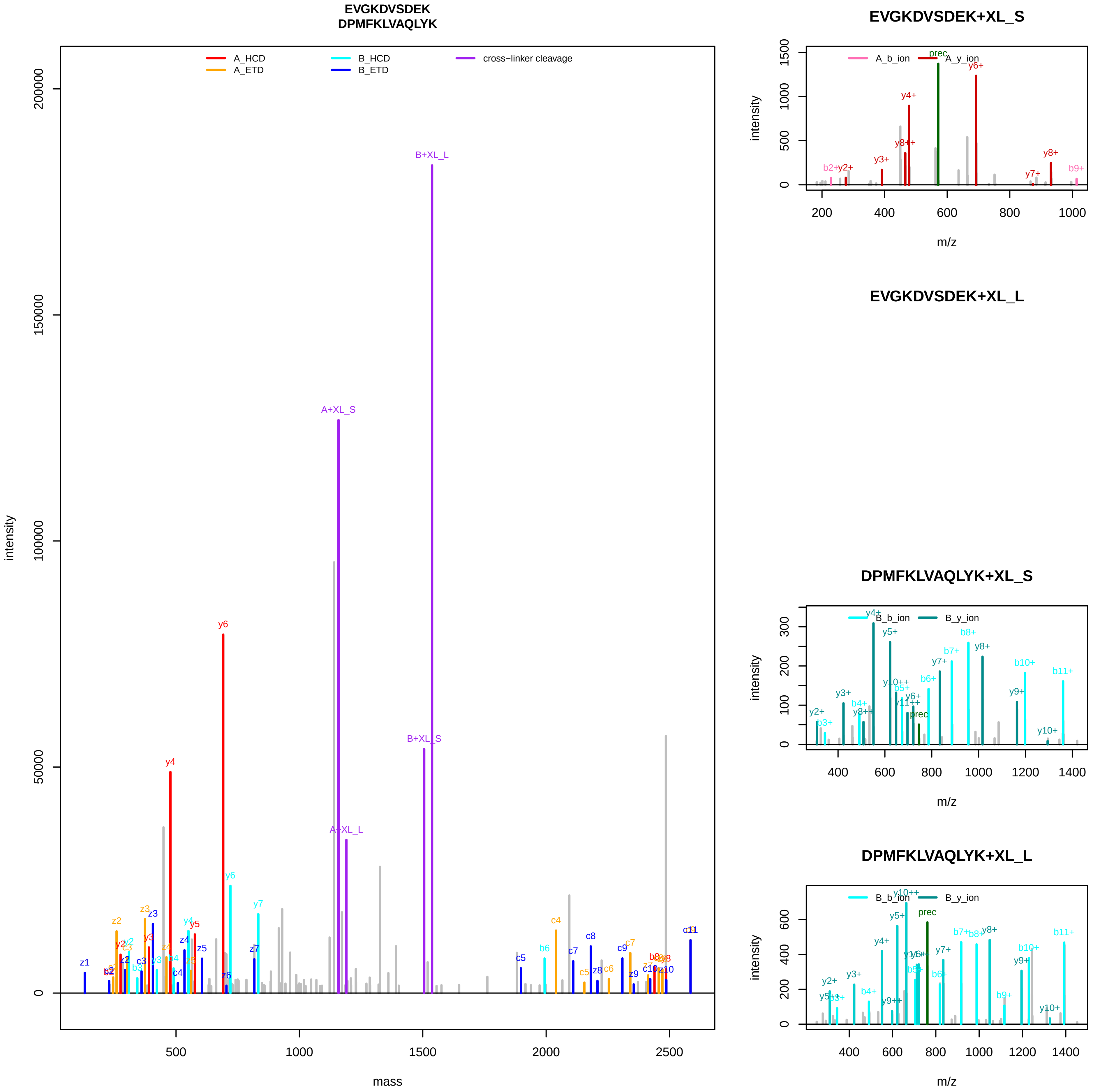
EVGKDVSDEK
DPMFKLVAQLYK
A_HCD
A_ETD
B_HCD
B_ETD
cross−linker cleavage
B+XL_L
A+XL_S
B+XL_S
A+XL_L
y4
y5
y6
y7
z1
z2
z3
z4
z5
z6
z7
z8
z9
z10
c3
c4
c5
c6
c7
c8
c9
c10
c11
b6
b8
y8
intensity
mass
0
50000
100000
150000
200000
500
1000
1500
2000
2500
EVGKDVSDEK+XL_S
A_b_ion
A_y_ion
prec
b2+
y2+
y3+
y4+
y6+
y7+
y8+
y8++
b9+
intensity
m/z
0
500
1000
1500
200
400
600
800
1000
EVGKDVSDEK+XL_L
DPMFKLVAQLYK+XL_S
B_b_ion
B_y_ion
prec
y2+
b3+
y3+
b4+
y4+
y5+
b5+
y6+
b6+
y7+
b7+
b8+
y8+
y8++
y9+
b10+
y10+
y10++
y11++
b11+
intensity
m/z
0
100
200
300
400
600
800
1000
1200
1400
DPMFKLVAQLYK+XL_L
B_b_ion
B_y_ion
prec
y2+
y3+
b3+
b4+
y4+
y5+
b5+
b6+
y6+
y7+
b7+
b8+
y8+
b9+
y9+
y9++
b10+
y10+
y10++
b11+
intensity
m/z
0
200
400
600
400
600
800
1000
1200
1400
